# Rotating Disk Electrodes beyond the Levich Approximation: Physics-Informed Neural Networks Reveal and Quantify Edge Effects

**DOI:** 10.1021/acs.analchem.3c01936

**Published:** 2023-08-17

**Authors:** Haotian Chen, Enno Kätelhön, Richard G. Compton

**Affiliations:** † Department of Chemistry, Physical and Theoretical Chemistry Laboratory, Oxford University, South Parks Road, Oxford OX1 3QZ, Great Britain; ‡ Accenture GmbH, Campus Kronberg, Kronberg im Taunus 61476, Germany

## Abstract

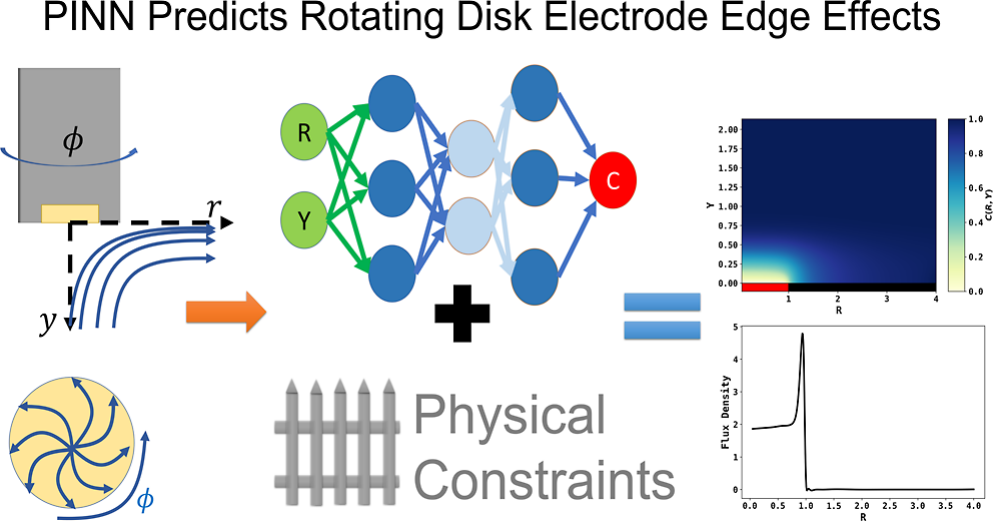

Physics-informed neural networks are used to characterize the mass transport to the rotating disk electrode (RDE), the most widely employed hydrodynamic electrode in electroanalysis. The PINN approach was first quantitatively verified via 1D simulations under the Levich approximation for cyclic voltammetry and chronoamperometry, allowing comparison of the results with finite difference simulations and analytical equations. However, the Levich approximation is only accurate for high Schmidt numbers (*Sc* > 1000). The PINN approach allowed consideration of smaller *Sc*, achieving an analytical level of accuracy (error <0.1%) comparable with independent numerical evaluation and confirming that the errors in the Levich equation can be as high as 3% when *Sc* = 1000 for rapidly diffusing species in aqueous solution. Entirely novel, the PINNs permit the solution of the 2D diffusion equation under cylindrical geometry incorporating radial diffusion and reveal the rotating disk electrode edge effect as a consequence of the nonuniform accessibility of the disc with greater currents flowing near the extremities. The contribution to the total current is quantified as a function of the rotation speed, disk radius, and analyte diffusion coefficient. The success in extending the theory for the rotating disk electrode beyond the Levich equation shows that PINNs can be an easier and more powerful substitute for conventional methods, both analytical and simulation based.

## Introduction

Recent advances of physics-informed neural networks (PINNs),^1^ as a novel partial differential equation (PDE) solving tool, have started to transform many science and engineering disciplines.^2^ PINNs are a unified framework capable of both data-driven discovery of PDEs (the inverse problem) and, as addressed in this paper, data-driven solution of PDEs (the forward problem). Training a PINN can be considered an unsupervised strategy by encoding physical laws into the loss function of a neural network. For the forward problem, the PINN algorithm is essentially a mesh-free, multidimensional solver that finds the solution to a PDE by minimizing the aforementioned loss function. Notable examples include solving stiff chemical kinetics,^3,4^ non-Newtonian fluid modeling^5^ and simulating voltammetry in 1-D to 3-D electrode geometries,^6,7^ convective-diffusion mass transport, and/or nonlinear chemical kinetics.^8^ For the inverse problem, PINNs can accommodate noisy experimental data and make physically consistent predictions. For example, PINNs and their variant, extended PINNs (XPINNs), have solved inverse problems in supersonic flows considered impossible for standard numerical methods.^9^

Despite PINNs flourishing in fluid dynamics^10^ and science/engineering in general,^2^ their applications are still scarce in chemistry. We attribute this to the concern that PINNs are just easier but not more capable alternatives to traditional numerical methods. To address such concerns, we challenged PINN to unveil the edge effect on a rotating disk electrode (RDE). Until then, this problem was addressed using the singular-perturbation method by Newman and colleagues, but it was only able to provide a self-confessed (well-) educated “guess” as the solution.^11^ The complexity of the 2-D problem and the discontinuous boundary conditions have discouraged electrochemists from attempting simulations using finite difference or finite element methods. To date, Newman’s approach is still state-of-the-art and the best approximation.

**Figure 1:**
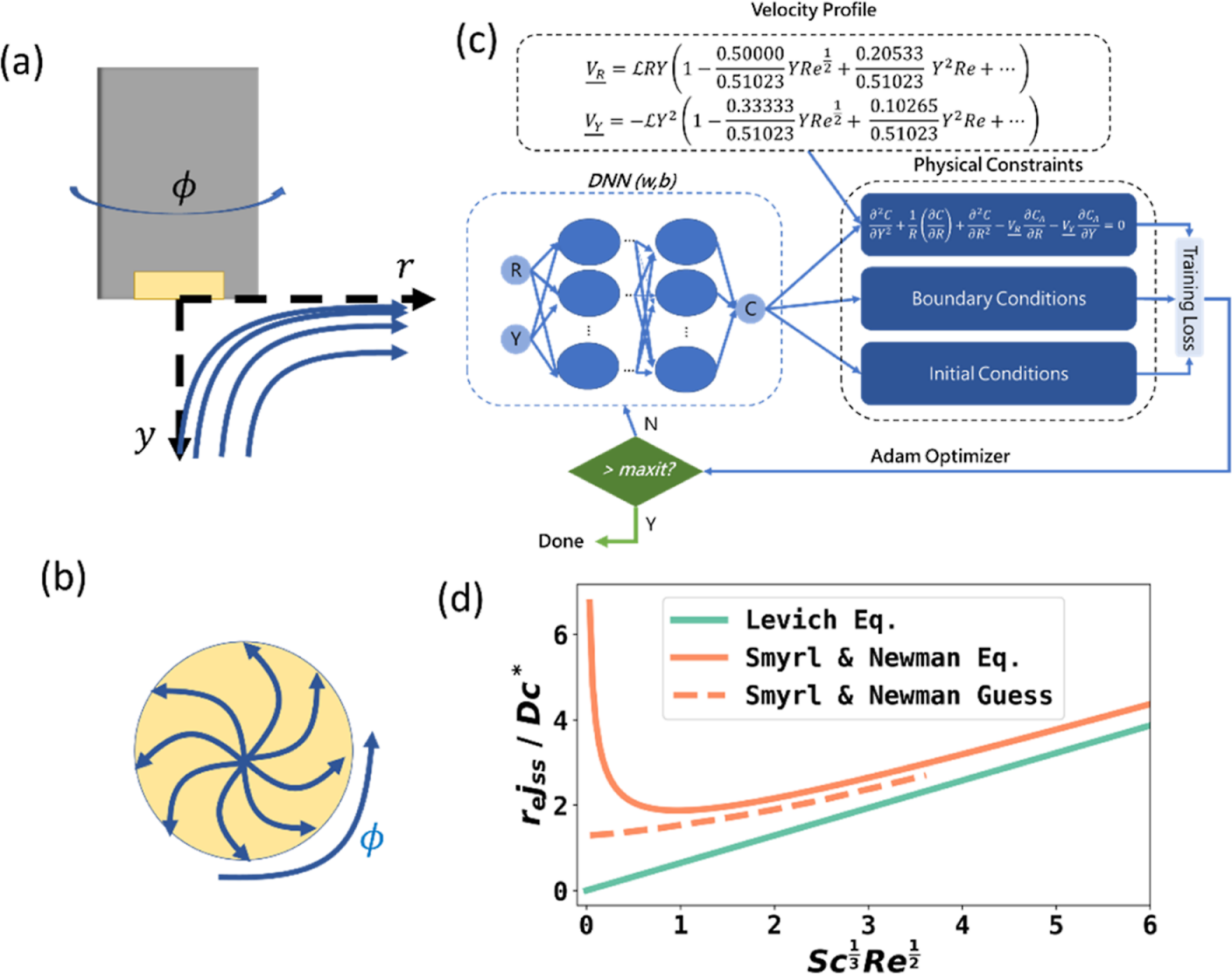
(a,b) Rotating electrode with a metal conductive area (golden). Blue arrow shows the hydrodynamic velocity in cylindrical coordinates, *r*, *y*, and ϕ. (c) Illustration of the PINN training to solve the 2-D steady state simulation at a RDE with multiple Schmidt number corrections. Notably, the prediction from a dense neural network is constrained by physical conditions, including PDEs, BCs, and ICs. (d) Analytical results obtained by Newman and colleagues in comparison with the Levich equation. Reproduced with permission from ref (11) Copyright 2017 IOP Publishing.

The rotating disk electrode is the most popular hydrodynamic electrode and is widely used by electro-analysts as well as fundamental electrochemists, not least since the device provides a well-defined convective flow of solution (analyte) to the electrode surface, allowing steady-state measurements.^12^ A schematic flow profile is shown in Figure 1a,b and the resulting diffusion-controlled currents have long been known, arising from the theoretical work of Levich^13^ and rigorously validated experimentally by the Frumkin School of Electrochemistry.^14^ In particular, Levich established the following equation for the transport-controlled steady-state current and diffusion layer thickness at the rotating electrode
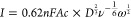

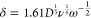
1where the symbols are defined in Table 1. The derivation of eq 1 assumes that the disk is uniformly accessible, implying that convection diffusion normal to the electrode is only considered so that the problem becomes one-dimensional. The Levich approach uses an approximate equation describing the solution velocity as a function of the distance away from the electrode under laminar hydrodynamic conditions, as evaluated by Kármán^15^ and Cochran^16^ retaining only the first term in a series expansion. This approximation is valid for large Schmidt numbers, *Sc*, where the diffusion layer due to electrolysis is small in scale compared to the hydrodynamic layer established by the rotating electrode.

**Table 1:** Definition of the Dimensional Parameters

variable(s)	symbol(s)	definition(s)	unit
concentration and bulk concentration of any species *j*	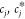		mol/m^3^
diffusion coefficient of any species *j*	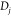		m^2^/s
applied potential and formal potential	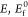		V
frequency of rotation	*f*		Hz
kinematic viscosity	ν (Greek letter)		m^2^/s
scan rate	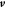 (English letter, bold)		V/s
fluid velocity in (*x*, *y*, and *z*) direction	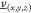		m/s
fluid velocity in the *y* direction with Levich approximation	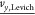	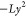	m/s
approximate fluid velocity in the *r* direction	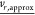	*Lyr*	m/s
rotation speed	ω	2π*f*	rad/s
Reynolds number	*Re*	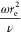	dimensionless
Schmidt number	*Sc*	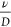	dimensionless
scaling factor for ν	*L*	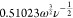	m^–^^1^ s^–^^1^

The assumption that the rotating electrode is uniformly accessible is strictly correct only in the limit of an electrode of infinite radius. Finite electrodes will show edge effects resulting from radial diffusion, and these effects will become increasingly large at slower rotation speeds where, in the limit of a static disc electrode, the diffusional transport becomes fully convergent and the steady-state diffusion limited current is described as follows
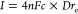
2where the diffusion layer is approximately hemispherical in shape rather than planar. The consequence of the edge effect at the finite rotating electrode is to (slightly) destroy the perfect uniform accessibility of the infinite rotating disc, leading to a greater local current density at the disc edges as compared to the disk center. This causes the overall disk current to increase a little above that predicted by the Levich equation.

The primary aim of this paper is to use PINNs to quantify this small enhancement (see Figure 1c) noting that an approximate analytical result was obtained by Newman,^11^ which predicts an asymptotic approach to the Levich equation as shown in Figure 1d, where the *x* axis is *Sc*^1/3^*Re*^1/2^ (proportional to ω^1/2^). Recognizing the limitations of their approach, which becomes increasingly obvious for smaller rotation speeds, Newman offered the displayed “guess” (his description), reflecting his physical and mathematical intuition. Note that the intercept on the *y* axis corresponds to eq 2. The problem is important so as to define the electrode geometry and rotation speed and the solute diffusion coefficients for which the Levich equation applies quantitatively.

We show below that the PINN approach quantifies the edge effect and, furthermore, allows easy comparison with the deviation caused by the high Schmidt number correction, with the latter shown to be the more significant effect for most systems. The two factors act in opposite directions on the Levich-predicted current but do not cancel. All simulation programs are available on https://github.com/nmerovingian/PINN4RDE with neural network weights for users’ convenience.

## Theory

In the following, the electrochemical reaction is assumed to be a simple one-electron reduction of the form of A + e ⇄ B, where A and B are stable solution phase species. We assume that there is only A in bulk solution, so that *c*_B_^*^ = 0 and that A and B have similar diffusion coefficients, *D*_A_ = *D*_B_; we model only species A since the concentration of species B is readily deduced via *c*_A_ + *c*_B_ = *c*_A_^*^. Sufficient electrolyte ensures that the mass transport is exclusively via diffusion and forced convection. To validate simulations using PINNs, results are compared with finite difference simulations,^17^ analytical expressions, and/or literature values where applicable.

### 1-D Simulation of RDE

Assuming a uniformly accessible RDE under laminar flow conditions and neglecting edge effects, the mass transport equation is simplified as one-dimensional
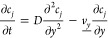
3where  is given in the literature as follows^15,16^
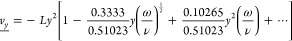
4

In the Levich approximation,  is approximated as , where *L* is defined in Table 1. Additional correction terms are shown in the Schmidt number correction section in Supporting Information. Physically, this is valid when the diffusion layer thickness is much less than the scale of the hydrodynamic layer established by the rotating electrode. The boundary conditions for the simulation of electrochemically reversible (“Nernstian”) linear sweep voltammetry at a RDE are
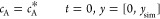
5
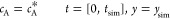
6
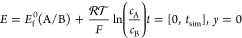
7where *y*_sim_ is the outer boundary of the simulated space and *t*_sim_ is the timespan of the voltammetry experiment. *E* and *E*_f_^0^ are the applied potential and the formal potential of the couple, respectively.  and *F* are the gas constant, temperature, and the Faraday constant. Equation 7 is the Nernst equation. Alternatively, for simulating the steady-state limiting current, it is replaced by *c*_A_ = 0, and the time derivative in eq 3is equated to zero.

### Steady-State 2-D Simulation of the Rotating Disk Electrode

When the edge effect is not negligible, notably at a low rotational frequency, the RDE simulation becomes two-dimensional, and the mass transport equation at the steady state is
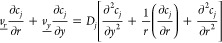
8where  is defined as
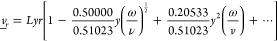
9

Close to the electrode  is approximated as . Under steady state conditions, the mass transport equation is time independent, and the boundary conditions corresponding to the transport limited current are
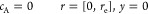
10
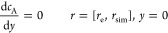
11
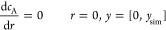
12
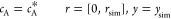
13
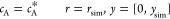
14

### Dimensionless Variables

Electrochemistry simulations commonly adopt dimensionless parameters for multiple advantages. First, dimensionless parameters increase the generality of the simulations, avoiding the need for repetitive simulations for different parameters; in addition, they reduce round-off errors during simulation, as all quantities are near the order of unity. In the context of PINN training, dimensionless PDEs are favorable to the optimizer^18^ and crucial to avoid the exploding/diminishing gradient problem.^19^

Using the dimensionless parameters defined in Table 2, the transformed mass transport equation for 1-D simulation is
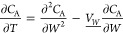
15where  is defined as
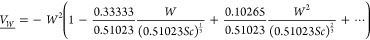
16

**Table 2:** Definition of Dimensionless Parameters^a^

parameter	dimensionless form
concentration	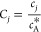
diffusion coefficient	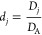
spatial coordinate in the *y* direction for 1-D simulation	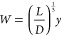
spatial coordinate in the *y* direction for 2-D simulation	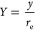
spatial coordinates in the *r* direction for 2-D simulation	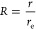
fluid velocity in the *W* direction under the Levich approximation	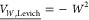
approximate fluid velocity in the *R* direction	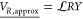
fluid velocity in the *Y* direction under the Levich approximation	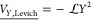
time	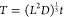
dimensionless auxiliary variable	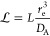
potential	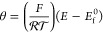
scan rate	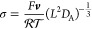
flux	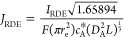

a *F*,  and  are the Faraday constant, the gas constant, and temperature, respectively. *E* and *E*_f_^0^ are the potential and the formal potential, respectively.

For the 2-D simulation of RDE at steady state, the dimensionless mass transport equation becomes
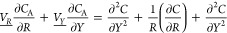
17where  and  are defined as
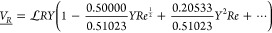
18
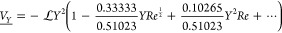
19

### PINN Simulation

PINN simulations of a 1-D RDE require prediction of the concentration *C*_A_ (*T*,*W*) as a function of the dimensionless coordinate *W* and time *T*. The PDE, initial, and boundary conditions for electrochemically reversible voltammetry to be solved by the PINN are
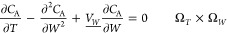
20
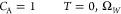
21
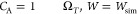
22
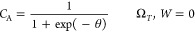
23where eq 20 represents the PDE embedded in PINN using the automatic differentiation (AD) utility, eq 21 is the initial condition, and eqs 22 and 23 are the boundary conditions. *W*_sim_ is the outer boundary of the simulation, equal to six times the Levich diffusion layer thickness.

Conventional data-driven neural networks are trained with labeled data to map the correlation between inputs and outputs. The PINN, on the contrary, encodes PDEs into its loss function to solve them by minimizing losses on large sets of collocation points. To enforce eq 20, for example,  collocation points  are generated using a uniform random distribution on the Ω_*T*_ × Ω_*W*_ domain as training inputs. The PINN framework has two parts. The first part is similar to a conventional data-driven neural network that takes (*T*_*i*_,*W*_*i*_) as input and gives *C*_A_ (*T*_*i*_,*W*_*i*_) as an output. The second part of the framework uses AD to calculate the derivatives of outputs respective to inputs and incorporate them into a loss function in the form of the mean square error (MSE) of the residuals
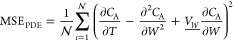
24

Equations 20–22 are encoded to the neural network in a similar fashion, allowing a linear combination of the MSE of the governing equations and initial and boundary conditions as the overall loss
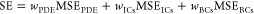
where *w* is the weight of each MSE function to balance their importance during training and the IC and BC subscripts refer to initial and boundary conditions, and a scheme of the setup is shown in Figure 1c. Although loss weights may require special attention,^20,21^ during the PINN simulation of RDE, equal weights for all losses provide satisfactory results, possibly because of the adoption of dimensionless parameters. The optimizer used for training was Adam^22^ (learning rate = 10^–3^) and the activation function for all hidden layers was hyperbolic tangent (tanh). During simulations,  was set between 1 and 3 million.

The dimensionless coordinates (*R* and *Y*) for the 2-D simulation are normalized against the radius of the electrode (*r*_e_) such that the dimensionless electrode radius is always unity, so that the neural network can “focus” around *R* = 1 to better resolve edge effects. In addition, curriculum learning was applied to train the PINN from high *Sc*^1/3^*Re*^1/2^ values to low values, to increase resolving power, and to facilitate convergence.^23^

The governing 2-D steady-state equation poses a serious challenge to a PINN: as , the exploding gradient problem will arise when *R* approaches zero. One possible solution is to upgrade the simulation to 3-D Cartesian coordinates at a cost of significant computational overhead.^7^ Instead, we employ a “divide-and-conquer” strategy, assuming that a small area at *R* ∈ [0,*R*_0_] is unaffected by the edge effect, such that the concentration profile at steady state can be approximated using the analytical equation at a very large Schmidt number such that  where Γ is the regularized lower incomplete gamma function.^24^ The value of *R*_0_ is a hyperparameter, and *R*_0_ = 0.05 is used throughout the simulations reported. The domain *R* ∈ [*R*_0_,*R*_sim_] is solved with the PINN. The equation set is
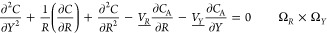
25
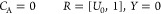
26
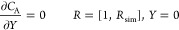
27
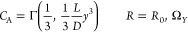
28
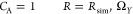
29
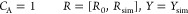
30where *R*_sim_ and *Y*_sim_ are the outer boundaries of simulation equal to four times the dimensionless diffusion layer thickness such that Ω_*R*_ ∈ [*R*_0_,*R*_sim_] and Ω_*Y*_ ∈ [0,*Y*_sim_]. After training, a concentration profile in the two-dimensional domain is obtained, and the flux on the electrode surface is directly given by the neural network using automatic differentiation
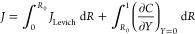
31

The theory and derivation of the FD method for 1-D simulation of RDE are provided in the Finite Difference Simulation of Rotating Disk Electrode section in the Supporting Information. The structure and workflow for PINN 2-D simulation of RDE are provided in the PINN Architecture section in the Supporting Information. A glossary for PINN is also provided in the Supporting Information. The sensitivity of hyperparameters, including the outer boundary of the simulation, and *R*_0_ is provided in the Hyperparameter Sensitivity section in the Supporting Information.

### Simulation Methods

The PINN simulation programs were written with Python, and PINN algorithms were implemented using TensorFlow 2.3. A 1-D finite difference simulation of cyclic voltammetry at a RDE was solved using the implicit Euler method and implemented in Python. The tridiagonal matrix was solved using the Scipy package. Note that for 2-D steady-state simulations and 1-D time-dependent simulations, it took less than 25 min to converge using 15 CPU cores without a GPU on the advanced research computing (ARC) cluster at the University of Oxford. The relatively low hardware requirement (as no GPU was used) and fast convergence speed using PINN signal the applicability of high-throughput simulations compared to other numerical methods.

## Results and Discussion

First, PINN simulations for cyclic voltammetry and chronoamperometry at an RDE with the Levich approximation are reported. These are compared with FD and/or literature reports, thus validating the approach. Second, Schmidt number corrections are added to the velocity profile so that the PINN can accurately solve the steady state flux at low *Sc* values, and the predicted flux is quantitatively compared with the literature. Third, the PINN approach was further progressed to novelly solve edge effects at an RDE at slow rotational frequency by employing PINN 2-D steady state simulations. Lastly, Schmidt number corrections are shown to be easily implemented for 2-D simulations. To convert between dimensional and dimensionless variables, the following variables are assumed, unless otherwise specified: *f* = 50 Hz, *D* = 10^–9^ m^2^ s^–1^, ν = 10^–6^ m^2^ s^–1^, *r*_e_ = 10 μm at 298 K.

### 1-D Simulation of Cyclic Voltammetry and Chronoamperometry

**Figure 2:**
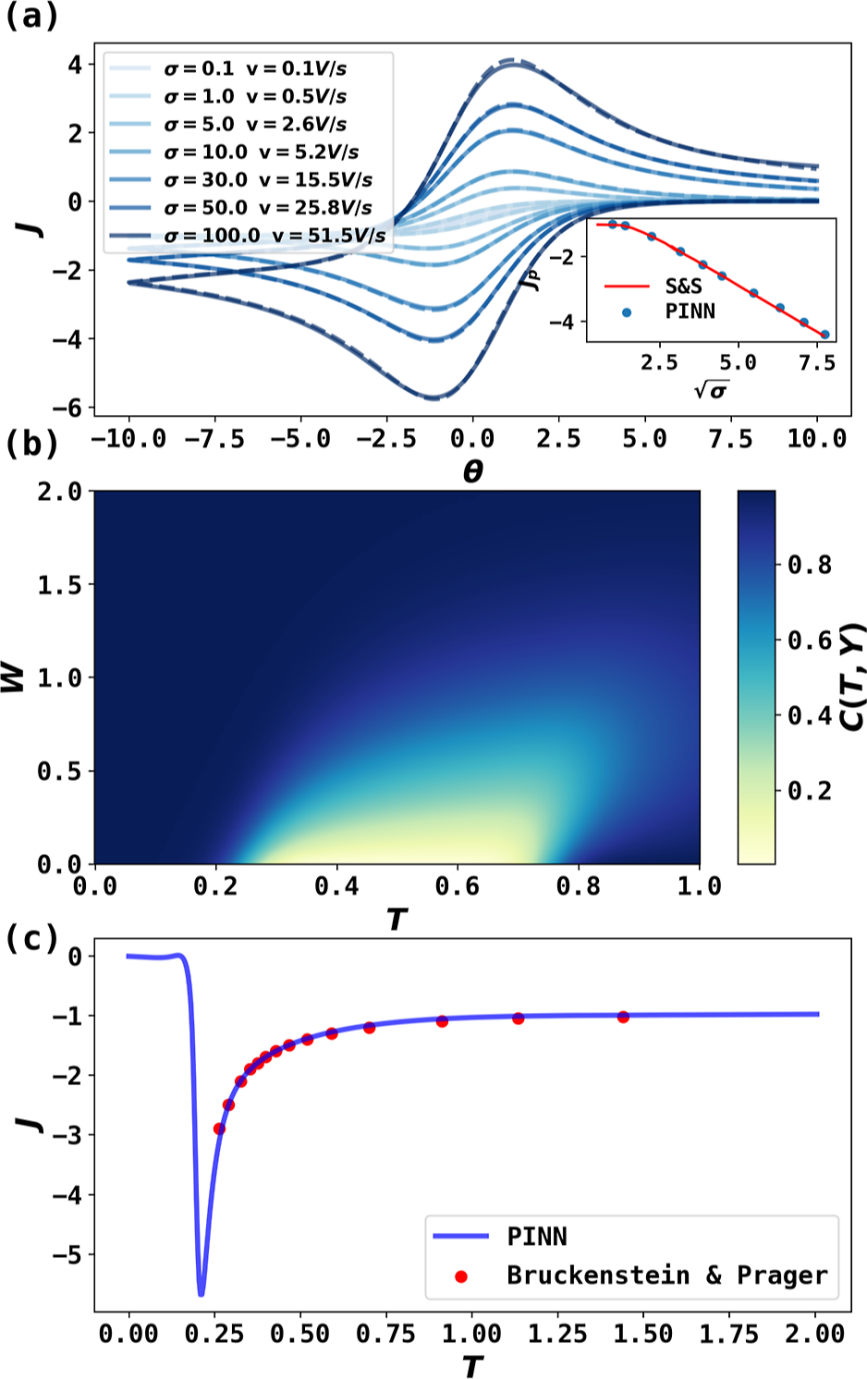
(a) Cyclic voltammetry at a RDE predicted by PINN (solid lines) is compared with finite difference simulations (dashed line). The inset compares the forward scan peak flux predicted by PINN with the analytical equation established by Strutwolf and Schoeller.^25^ Both dimensionless and the corresponding dimensional scan rates are shown in the legend using the parameters mentioned in the text. (b) Time-dependent concentration profile predicted by PINN when σ = 40. The decreased concentration near the electrode surface between *T* = 0.25 and 0.75 represents electrochemical reaction at the electrode governed by the Nernst equation. (c) Potential step chronoamperogram predicted by PINN is validated with independent work from Bruckenstein and Prager.^26,27^ The excitation waveform was applied at *T* = 0.2, and a small positive peak before the sharp increase in flux magnitude was due to an artifact with the relaxation time before *T* = 0.2. The time interval between 0 and 0.2 is known as the conditioning time, where no electrochemical reaction happens.

First, PINN simulations of electrochemically reversible cyclic voltammetry using the Levich approximation were performed at different dimensionless scan rates from σ = 0.1 to σ = 100, corresponding to *v* from 0.1 to 51.5 V/s and validated with FD simulation and independent simulation results from Strutwolf and Schoeller after adjusting for different normalizations of scan rates.^25^ The PINN (solid lines) and FD (dashed lines) simulations are overlaid in Figure 2a, showing a good agreement between the two methods, and the PINN simulated peak fluxes agree well with results reported by Strutwolf and Schoeller, as shown in the inset of Figure 2a. Figure 2b shows the concentration profile at σ = 40. Potential step chronoamperometry at a RDE was also simulated using PINN and compared with an independent simulation reported by Bruckenstein and Prager^26^ and shown in Figure 2c. Note that a conditioning time is adopted such that the excitation waveform is applied after *T* > 0.2.

The good agreement of PINN with analytical and/or FD simulations proves the viability of using PINN to simulate hydrodynamic voltammetry at the RDE. The quantitative success encourages the exploration of more accurate (but more complex) governing equations, to which we next turn.

### Schmidt Number Corrections in the 1D Simulation of the RDE

**Figure 3:**
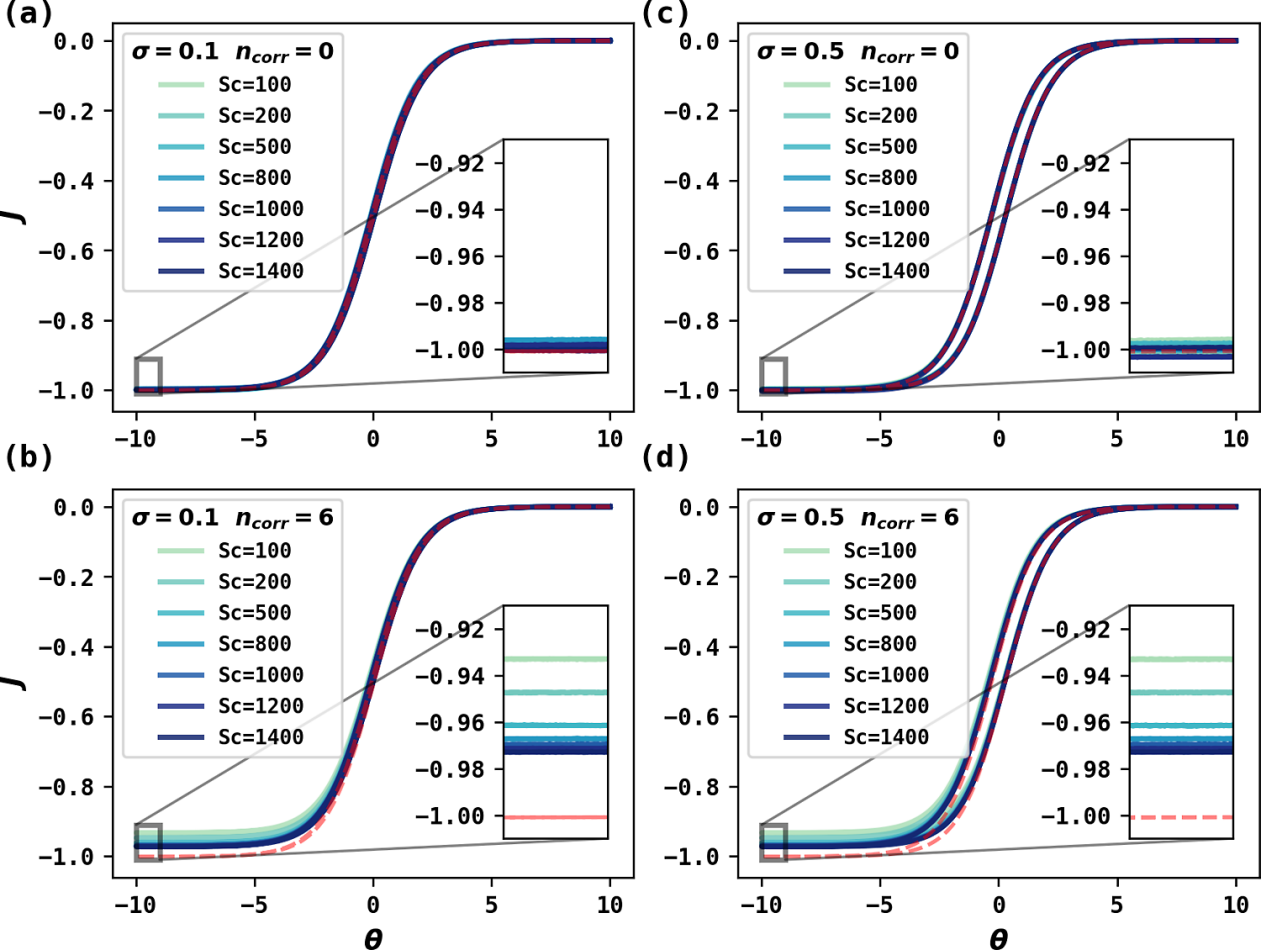
PINN simulation of voltammetry at RDE at (a,b) σ = 0.1 and (c,d) σ = 0.5, corresponding to ***v*** = **0.05** and **0.25 V/s** when *Sc* = 1000, and increasing number of Schmidt number correction terms. There are 0 or 6 correction terms in (a,c) or (b,d), respectively, at different Schmidt numbers (*Sc* = 100 to 1400). The red-dashed curve in each plot represents the Levich prediction without considering Schmidt number corrections.

We next consider the inclusion of additional Schmidt number correction terms to the fluid velocity profile in eq 9 for the PINN simulation, as the Levich approximation becomes increasingly poor for decreasing Schmidt numbers when *Sc* < 1000. Specially, the Levich approximation introduces 7.2 or 3.1% error when *Sc* = 100 or 1000.^28^ Two Schmidt number correction terms are included in PINN as shown in eq 16 to facilitate comparison with the literature solution employing the same corrections.^28^ PINN simulations were performed at 7 different *Sc* values from 100 to 1400 at different dimensionless scan rates (σ = 0.1, 0.5) and up to 6 correction terms. The corresponding dimensional scan rates are 0.05 and 0.25 V/s when *Sc* = 1000. The simulation results are shown in Figure 3. Each column (a–d) suggests that with Schmidt number corrections (*n*_corr._ > 0), the magnitude of limiting flux at smaller *Sc* values increasingly deviates from the prediction using the Levich approximation. The two scan rates for each column suggest that PINN can solve more complicated time-dependent problems without restriction to steady state conditions.

Noticeably, the Schmidt number correction terms are easily introduced into the PINN through an auxiliary input without much computational overhead, while retaining the option to easily incorporate more correction terms when necessary. The simulation program provided in the public repository (see Introduction) has implemented six correction terms, providing redundant flexibility in case of need. Figure S1 illustrates the difference in concentration profile with two or no correction terms (*C*_*n*_corr_ = 2_ – *C*_*n*_corr_ = 0_) and shows a slightly more depleted mass transport layer near the electrode surface. The PINN simulation results are then compared with the numerical solution (which used two correction terms) reported in literature,^28^ and tabulated in Table 3. Table 3 suggests that three corrections are sufficient to capture the steady state flux, as the effect of the fourth term and beyond is negligible for the Schmidt numbers considered. Note that the effect of the extra convective terms normal to the electrode is to reduce the current as compared to the predictions of the Levich approach.

**Table 3:** Limiting Flux Simulated Using PINN with Up to Six Schmidt Number Corrections When σ = 0.1^a^

*Sc*	PINN *n*_corr._ = 1	PINN *n*_corr._ = 2	PINN *n*_corr._ = 6	numerical solution	absolute error, %
100	–0.9222	–0.9336	–0.9330	–0.9332	0.04
200	–0.9413	–0.9477	–0.9474	–0.9476	0.01
500	–0.9588	–0.9614	–0.9614	–0.9615	0.01
800	–0.9649	–0.9674	–0.9673	–0.9672	0.02
1000	–0.9680	–0.9699	–0.9697	–0.9696	0.03
1200	–0.9699	–0.9715	–0.9714	–0.9714	0.01
1400	–0.9720	–0.9724	–0.9729	–0.9729	0.05

a The numerical solutions were calculated with two corrections, thus compared with the PINN simulation with two corrections and the absolute error in comparison with independent numerical simulations^28^ is reported in the last column.

We consider an aqueous (298 K) kinematic viscosity of 10^–6^ m^2^ s^–1^ and a typical aqueous solution-based diffusion coefficient of 10^–9^ m^2^ s^–1^ such that *Sc* = 1000, neglecting edge effects. The Schmidt number of corrected current is 3.04% below the Levich equation, a significant number in the context of analytical chemistry. In the case of lower kinematic viscosity and a higher diffusion coefficient, the deviation will be more significant. For example, a low Schmidt number was reported for gas transport in salt water, suggesting the experimental relevance and importance of Schmidt number corrections.^29^

As shown in Table 3, the average absolute error between a full numerical solution and the PINN prediction is as low as 0.02%, suggesting that PINNs can achieve an analytical level of accuracy while easily implementing 6, and likely more, Schmidt number corrections into the governing PDEs. This scenario proves the practicality of PINN to solve time-dependent nonlinear PDEs for chemistry, with the possibility of outdating traditional numerical methods.

### Unveiling the Edge Effect at the RDE

**Figure 4:**
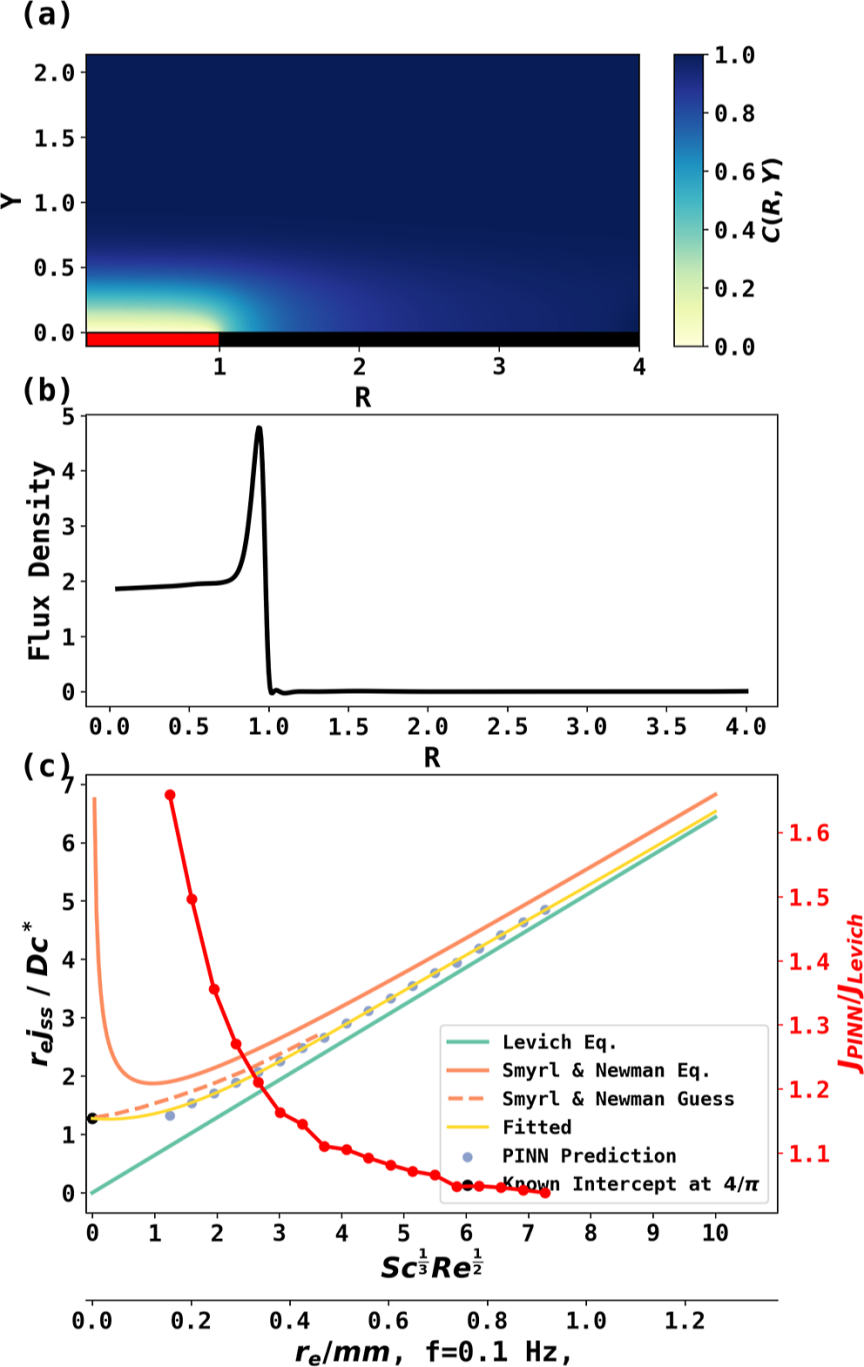
(a) Steady-state concentration profile predicted by PINN when *Sc*^1/3^*Re*^1/2^ = 3.01. Red and black rectangles represent the electrochemical active electrode and insulating materials, respectively, and (b) flux density at the electrode surface, which shows significant edge contribution near *R* = 1. (c) Comparing the PINN prediction (blue scatter points from *Sc*^1/3^*Re*^1/2^ = 1.24 to 7.27), Smyrl and Newman’s work (orange solid and dashed lines) and the Levich equation’s (green line) prediction of the steady-state flux at different dimensionless rotation rates, *Sc*^1/3^*Re*^1/2^. PINN prediction was fitted (yellow line) along with a known intercept at  to give a general expression of peak current as . The right-hand *y*-axis and red line with markers present the ratio of the PINN-predicted current (with edge effect) to the relative Levich prediction. The lower *x*-axis provides guidance in real experiments, assuming a rotational frequency of 0.1 Hz. Note that when *Sc*^1/3^*Re*^1/2^ < 1.24, it is not possible to distinguish the currents at a rotating disk from a stationary disk; the designed PINN is not designed to solve the stationary disk problem.

Edge effects at the RDE, which are not negligible at slow rotational speeds, necessitate a 2-D simulation. First, we report PINN simulations at steady state at a RDE at different dimensionless frequencies (*Re*^1/3^*Sc*^1/2^) by employing PINN to directly solve the convective-diffusion to a RDE without recourse to mathematical approximations. The velocity profiles in both *y* and *r* directions are first described with the Levich equation (i.e., no correction terms) to allow comparison with the literature,^11^ and then simulated with additional correction terms as shown in the next section. Figure 4 gives an example of the steady-state concentration profile at (*Re*^1/3^*Sc*^1/2^ = 3.01) and the convergent mass transport to the disk where *R* > 1, such that an enhanced flux density is observed near the electrode edge as shown in Figure 4b (the “edge effect”). Figure 4c visualizes the PINN predictions of the steady state flux, the Newman analytical equation and guesses, and the Levich equation. PINN-predicted fluxes are in between the estimates of the Newman equation and the Levich equation. The red curve with markers (note especially the right-hand *y*-axis) suggests a larger deviation from Levich equation at a lower rotational frequency. For example, when *Sc*^1/3^*Re*^1/2^ < 3, the edge effect enhances the flux by more than 50%. The second *x*-axis provides an estimate, as when the rotational frequency is 0.1 Hz, significant edge effects can be observed if *r*_e_ < 0.5 mm. The critical rotational frequency below which the edge effect causes more than 1% deviation from the Levich equation is estimated at different electrode radii and reported in the Significance of the Edge Effect section in the Supporting Information. For example, for an electrode with a 100 μm radius, the critical frequency is 15.4 Hz.

To provide an estimate of the edge effect at different rotational frequencies without further training, a curve was fitted to the following equation based on PINN simulation results and a known intercept, and the fitted equation and parameters are
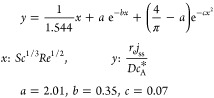
32where  is the steady state flux predicted by the Levich equation, and the two additional terms following it present addition flux contributed by the edge effect. The fitting equation is enforced with a known intercept at , and the fitted curve is shown in Figure 4c.

The fitted curve quantifies the edge effect at an RDE using a more definitive and systematic approach than was available to Newman. By incorporating complicated PDEs into loss constraints, PINN provides a shortcut to solve chemistry problems that conventional methods are incapable of.

### 2D Simulation of RDE with Schmidt Number Corrections

**Figure 5:**
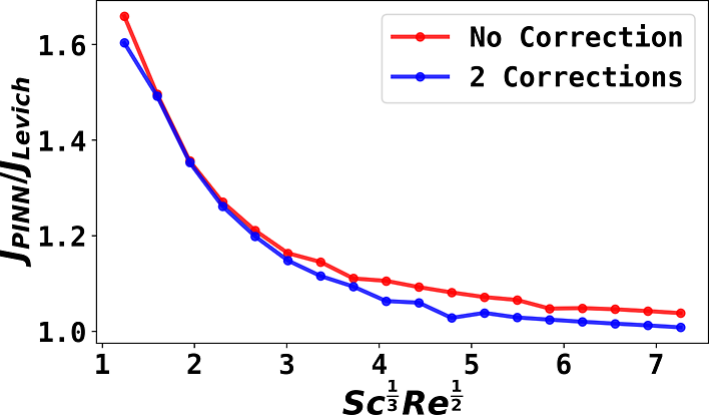
Comparing steady-state flux for 2-D simulation at a RDE at different dimensionless rotational speeds with no Schmidt number correction (red) and with two Schmidt number corrections (blue). The corrections are in both the *R* and *Y* directions. The reported flux (*J*_PINN_) is normalized against the Levich equation (*J*_Levich_).

To fully exploit the capability of the PINN approach to solve complicated hydrodynamic problems, two Schmidt correction terms (see eqs 18 and 19) in both *R* and *Y* directions are incorporated in the velocity profiles for 2-D steady state simulations at different dimensionless rotational frequencies (*Sc*^1/3^*Re*^1/2^) and shown in Figure 5. The steady-state fluxes are lower, with two additional correction terms. Using this example, we therefore used PINN to solve a multidimensional PDE with complex nonlinear corrections as the governing equation. The problem is solved quantitatively with PINN for the first time, to the best of the author’s knowledge. We conclude that PNN, as an emerging discretization-free framework, may advance chemistry to solve harder problems, including hitherto insoluble problems, with relative ease. In addition, the computational time is reasonably fast: on a 20-core workstation, each 2-D simulation takes less than 30 min.

## Conclusions

We implemented PINNs to quantify convective-diffusion mass transport to a rotating disk electrode (RDE), first approximately in 1-D to mirror the Levich approach and then in 2-D to include radial diffusion effects, to evaluate a long-lasting challenge in computational electrochemistry, namely the edge effect at the rotating disk.^11^ Specifically, we rigorously introduced the background to Newman’s approach and his “guessed” solution, the mathematical formulations, and underlying principles of PINNs, followed by the PINN solution of these problems. First, PINN 1-D simulations of voltammetry and chronoamperometry were validated with the finite difference method and independent reports where applicable. Second, up to six Schmidt number corrections were successfully introduced to the 1-D simulations. Using PINNs, we report the ease of novelly implementing up to six nonlinear correction terms to solve time-dependent problems while achieving an analytical level of accuracy (absolute error ≈ 0.02%). Finally, we showed how a PINN quantifies the edge effects expected at an RDE for a slow rotational speed and, for the first time, provided a PINN data-generated working curve to quantify the deviation from the Levich equation. Lastly, Schmidt number corrections were also introduced in the 2-D simulations.

The work demonstrates the ability of PINN-based simulation approaches to solve multidimensional and highly complex PDEs in the context of electrochemistry. Results are achieved with high accuracy and relative ease, where the numerical method adopted by Smyrl and Newman has been challenged to solve the edge effect at RDE and stands in contrast to the common assumption that PINNs are solely a tool to produce data-driven estimates of the state of the system and its evolution rather than exact solutions to well-defined problems. While this is a relevant insight for itself, the understanding that PINNs have the capability of modeling complex electrochemical systems with relative ease and high accuracy points even further toward opportunities in electrochemistry beyond the here-presented modeling of RDEs and purely theoretical simulations, especially if the PINNs’ capability of highly accurate electrochemical modeling is combined with other capabilities of PINNs, in which the approach excels more classical approaches like finite element- or finite difference simulations. The latter often include the ability of PINNs to infer the state of a system and predict its temporal evolution even in scenarios where data is only available at a low quality, for instance, if it is affected by statistical fluctuations induced by the measurement process or if individual values are missing or false. The approach may hence be extended to the modeling of electrochemical systems other than RDEs that are governed by similar equations and where, at least in part, experimental data is used as a boundary condition to the simulation.

While the problem of modeling electrochemical systems based on limited or noisy data may be less common in academic research, where experiments can often be conducted under laboratory conditions at high accuracy, it is becoming increasingly important as an application in various industries. With the continuously more widespread use of Internet-of-Things (IoT) technology and cost-efficient methods to process large amounts of data, it has become possible and often economically viable to acquire and utilize data from individual electrochemical devices such as batteries or potentially fuel cells, even if these devices are to be connected in large numbers and their costs are low. A particularly relevant application is herein the development of battery digital twins,^30–32^ which may become an important tool in the management of batteries in electric vehicles. Approaches similar to the here-discussed PINNs approach may then act as a powerful analytics tool to understand processes within each of these electrochemical devices and to enable a bespoke operation strategy that is specific to an individual device, its usage, and its environment to enable a more sustainable and cost-efficient operation via accurate virtual representations of the physical assets and their functionality in the form of digital twins. In conclusion, we recommend using PINNs, not only as an easier but also a more capable alternative to traditional numerical methods for many convective-diffusion problems, and look forward to seeing them being used more widely in academic research and beyond.

## Supporting Information Available

Glossary; finite difference simulation of rotating disk electrode; Schmidt number corrections; concentration profile with/without Schmidt number corrections; PINN architecture; hyperparameter sensitivity; and significance of the edge effect. The code repository is available at https://github.com/nmerovingian/PINN4RDE(PDF)
