# Supplementary material for: Rotating Disk Electrodes beyond the Levich Approximation: Physics-Informed Neural Networks Reveal and Quantify Edge Effects
Source: Anal Chem. 2023 Aug 17;95(34):12826–34. doi: 10.1021/acs.analchem.3c01936 (PMC10469374; doi:10.1021/acs.analchem.3c01936)
Supplement: Supplementary file 1 — ac3c01936_si_001.pdf [file ac3c01936_si_001.pdf]

Supporting Information
Rotating Disk Electrodes Beyond the Levich Approximation: Physics-Informed Neural Networks Reveal and Quantify Edge Effects

Haotian Chen[a], Enno Kätelhön[b] and Richard G. Compton[a,*]

[a] Department of Chemistry, Physical and Theoretical Chemistry Laboratory, Oxford University, South Parks Road, Oxford OX1 3QZ, Great Britain

[b] Accenture GmbH, Campus Kronberg, 61476 Kronberg am Taunus, Germany

[*] Corresponding author. Email address: Richard.compton@chem.ox.ac.uk (R. G. Compton)

Table of Contents

1 Glossary 1
2 Finite Difference Simulation of Rotating Disk Electrode 2
3 Schmidt Number Corrections 3
4 Concentration Profile with/without Schmidt Number Corrections 4
5 PINN architecture 4
6 Hyperparameter sensitivity 6
7 Significance of the Edge Effect 7

1 Glossary

A table of glossary is added for readers unfamiliar with artificial intelligence and neural network terminologies.

Terms	Explanation
Partial Differential Equation (PDE)	PDEs are differential equations that compute a function of multiple variables
Ordinary Differential Equation (ODE)	ODEs are equations giving a function of only one variable
Artificial Neural Network (ANN)	ANNs are algorithms inspired by biological neural networks and comprise a network of interconnected nodes that make up a model. They can be understanded as statistical learning models used to estimate or approximate functions that depend on a large number of inputs and outputs.
Fully Connected Layer	Fully connected layer is a layer with weights and biases used to connect the neurons between two different layers. A neuron is a mathematical function that model the functioning of a biological neuron.
Fully Connected Neural Network	A fully connected layer refers to a neural network in which each neuron applies a linear transformation to that input vector through a weight matrix. Properly stacking multiple fully connected layers give a fully connected neural network.

Physics-Informed Neural Network (PINN)	A PINN is a type of universal function approximator that embeds knowledge of physical laws in the form of partial differential equations that govern a given data-set in the learning process.
Collocation Methods and Collocation Points	In mathematics, a collocation method is a method for numerical solution of ODEs, PDEs and integral equations. Collocation points are a number of points selected in the domain which satisfy the given equation at the collocation points.
Automatic Differentiation (AD)	AD is the technique to evaluate the partial derivative of a function specified by a computer program.
Loss Function	A loss function is a function that compares the target and the predicted output values; It measures how well the neural network models the training data.

2 Finite Difference Simulation of Rotating Disk Electrode

1-D simulations of rotating disk electrode (RDE) using PINNs were validated with finite difference simulations for the partial differential equation:

$$\frac{\partial C_A}{\partial T} = \frac{\partial^2 C_A}{\partial W^2} - \underline{V_{W,Levich}}\frac{\partial C_A}{\partial W} \tag{1}$$

where $\underline{V_{W,Levich}}$ is the velocity profile in the W direction where with Levich approximation $V_{W,Levich} = -W^2$. For a better resolution of the problem, Hale transformation[1] offers a convenient approach[2]:

$$U = \frac{\int_0^W \exp\left(-\frac{1}{3}W^3\right) dW}{\int_0^\infty \exp\left(-\frac{1}{3}W^3\right) dW} \tag{2}$$

such that the value $U = 0$ corresponds to electrode surface and $U = 1$ corresponds to a distance infinitely far from electrode. During simulation, the outer boundary of simulation is at $U_{sim} = 0.9999$, a distance sufficiently far away from electrode surface and unperturbed by surface reaction there. The boundary conditions after Hale transformation is:

$$C_A = 1 \qquad T = 0, U_{sim} \geq U \geq 0 \qquad 3.1$$
$$C_A = 1 \qquad T > 0, U = U_{sim} \qquad 3.2$$
$$C_A = \frac{1}{1 + \exp(-\theta)} \qquad T > 0, U = 0 \qquad 3.3$$

For simulation of linear sweep voltammetry, the dimensionless potential is linearly increased/decreased as a function of time and dimensionless scan rate σ as:

$$\begin{cases} 0 < T < T_{switch}, \theta = \theta_i - \sigma T \\ T = T_{switch}, \theta = \theta_{switch} \\ T_{switch} < T < 2T_{switch}, \theta = \theta_{switch} + \sigma(T - T_{switch}) \end{cases} \tag{4}$$

where θ_i is the forward scan starting potential and θ_{switch} is the reverse potential of triangular shape potential waveform and the direction of scan is switched when $T = T_{switch}$. To study reduction process, the sign of σ is positive, and vice versa for an oxidation process.

The problem was solved using backward implicit method[3] such that for species A at the spatial pint i and time point k, the solution is given by:

$$\frac{C_{A,i}^{k}-C_{A.i}^{k-1}}{\Delta T}=\frac{\exp\left(-\frac{2}{3}W_A^3\right)}{1.65894}\left(\frac{C_{A,i-1}^{k}-2C_{A,i}^{k}+C_{A,i+1}^{k}}{\Delta U^2}\right) \tag{5}$$

Solving an ordinary differential equation (ODE) can find the relationship between U and W as $\frac{dW}{dY}=\sqrt{1.65894}\exp\left(\frac{1}{3}W^3\right)$ and the boundary condition is $W_0=0$ corresponding to $Y=0$. During simulation, the ODE was solved using *odeint* function in *scipy*. Eq.5 can be rewritten as:

$$\alpha_{j,i}C_{j,i-1}^{k}+\beta_{j,i}C_{j,i}^{k}+\gamma_{j,i}C_{j,i+1}^{k}=\delta_{j,i}^{k} \tag{6}$$

where:

$$\alpha_{j,i}=-\frac{\exp\left(-\frac{2}{3}W_i^3\right)}{1.65894}\frac{\Delta T}{\Delta U^2} \tag{7}$$

$$\beta_{j,i}=2\frac{\exp\left(-\frac{2}{3}W_i^3\right)}{1.65894}\frac{\Delta T}{\Delta U^2}+1 \tag{8}$$

$$\gamma_{j,i}=-\frac{\exp\left(-\frac{2}{3}W_i^3\right)}{1.65894}\frac{\Delta T}{\Delta U^2} \tag{9}$$

$$\delta_{j,i}^{k}=C_{j,i}^{k-1} \tag{10}$$

Thus, the matrix to be solved iteratively during simulation is:

$$\begin{pmatrix}1&0&0&0&0&0&0&0&0\\ \alpha&\beta&\gamma&0&0&0&0&0&0\\ 0&\dots&\dots&\dots&0&0&0&0&0\\ 0&0&\alpha&\beta&\gamma&0&0&0&0\\ 0&0&0&\alpha&\beta&\gamma&0&0&0\\ 0&0&0&0&\alpha&\beta&\gamma&0&0\\ 0&0&0&0&0&\dots&\dots&\dots&0\\ 0&0&0&0&0&0&\alpha&\beta&\gamma\\ 0&0&0&0&0&0&0&0&1\end{pmatrix}\begin{pmatrix}C_0^k\\ C_1^k\\ \dots\\ C_{33}^k\\ C_{34}^k\\ C_{35}^k\\ \dots\\ C_{n-2}^k\\ C_{n-1}^k\end{pmatrix}=\begin{pmatrix}\frac{1}{1+\exp(-\theta)}\\ C_1^{k-1}\\ \dots\\ C_{33}^{k-1}\\ C_{34}^{k-1}\\ C_{35}^{k-1}\\ \dots\\ C_{n-2}^{k-1}\\ 1\end{pmatrix} \tag{11}$$

More information can be found in ref.[4]

3 Schmidt Number Corrections

For 1-D simulation of the RDE under laminar flow conditions, the velocity profile in the y-direction with 6 Schmidt number correction terms is:

$$\underline{v_y}=-Ly^2\left[1-\frac{0.3333}{0.51023}y\left(\frac{\omega}{\nu}\right)^{\frac{1}{2}}+\frac{0.10265}{0.51023}y^2\left(\frac{\omega}{\nu}\right)-\frac{0.01265}{0.51023}y^3\left(\frac{\omega}{\nu}\right)^{\frac{3}{2}}-\frac{0.00283}{0.51023}y^4\left(\frac{\omega}{\nu}\right)^2+\frac{0.00179}{0.51023}y^5\left(\frac{\omega}{\nu}\right)^{\frac{5}{2}}-\frac{0.00045}{0.51023}y^6\left(\frac{\omega}{\nu}\right)^3+\cdots\right] \tag{12}$$

For 2-D simulation, the additional velocity profile in the r-direction with 6 Schmidt number corrections is:

$$\underline{v_r} = Lyr\left[1 - \frac{0.50000}{0.51023}y\left(\frac{\omega}{\nu}\right)^{\frac{1}{2}} + \frac{0.20533}{0.51023}\,y^2\left(\frac{\omega}{\nu}\right) - \frac{0.03060}{0.51023}y^3\left(\frac{\omega}{\nu}\right)^{\frac{3}{2}} - \frac{0.0085}{0.51023}y^4\left(\frac{\omega}{\nu}\right)^2 + \frac{0.00628}{0.51023}y^5\left(\frac{\omega}{\nu}\right)^{\frac{5}{2}} - \frac{0.00180}{0.51023}y^6\left(\frac{\omega}{\nu}\right)^3 + \cdots\right] (13)$$

4 Concentration Profile with/without Schmidt Number Corrections

To visualize the effect of Schmidt number corrections on, the difference in concentration profile with/without two Schmidt number corrections when $Sc = 100$, $\sigma = 0.1$ was investigated. With the correction terms, a slightly more depleted concentration near the electrode surface is observed as shown in Figure S 1.

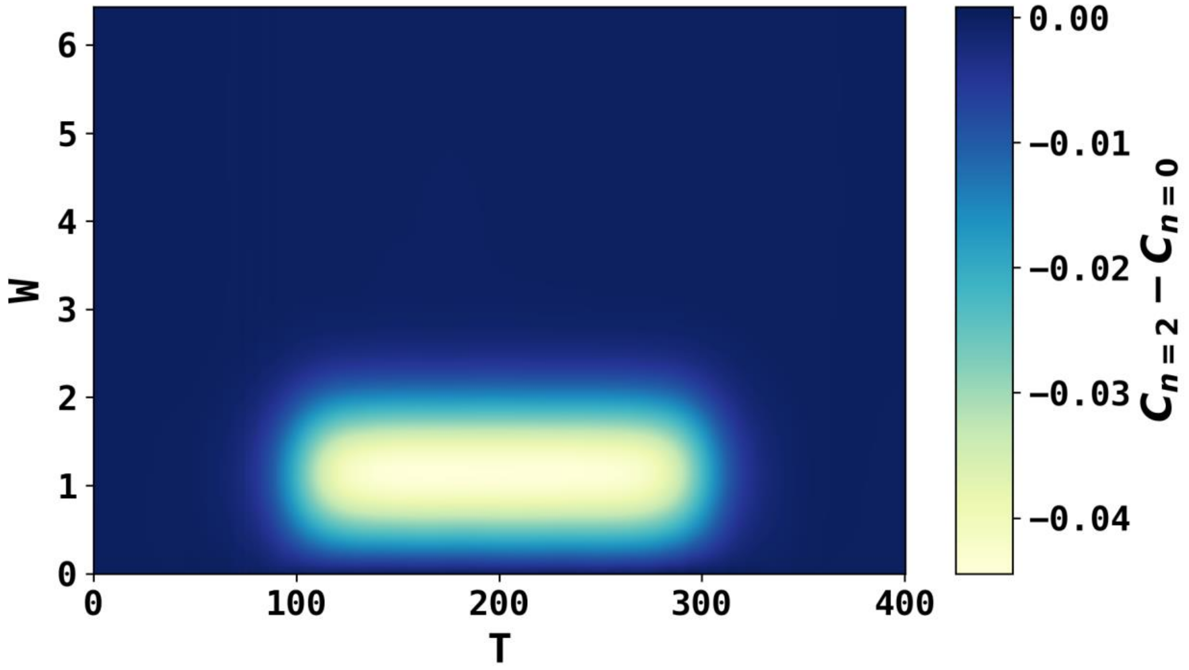

Figure S 1. Comparing the difference in concentration profile with/without two Schmidt number corrections when scan rate is fixed at $\sigma = 0.1$ and $Sc = 100$.

5 PINN architecture

The structure of the deep neural network backbone of PINN for 2D simulation at a RDE is shown in the table below along with the activation function, kernel initializer and the number of trainable parameters is tabulated in Table S 1. Figure S 2 illustrates the workflow of (a) generating training data, which are collocation points, feeding into a neural network and constraining with physical laws.

Table S 1. The structure of the neural network backbone for 2D simulation of RDE using PINN.

Layer (type)	Output Size	# Trainable Parameters	Notes
Input(Input Layer)	(batch size,3)	0	
Dense(Dense)	(batch size, 64)	256	activation='tanh', kernel_initializer='he_normal'

Dense_1(Dense)	(batch size, 32)	2080	activation='tanh', kernel_initializer='he_normal'
Dense_2(Dense)	(batch size, 32)	1056	activation='tanh', kernel_initializer='he_normal'
Dense_3(Dense)	(batch size, 32)	1056	activation='tanh', kernel_initializer='he_normal'
Dense_4(Dense)	(batch size, 64)	2112	activation='tanh', kernel_initializer='he_normal'
Dense_5(Dense)	(batch size, 1)	65	activation=None, kernel_initializer='he_normal'

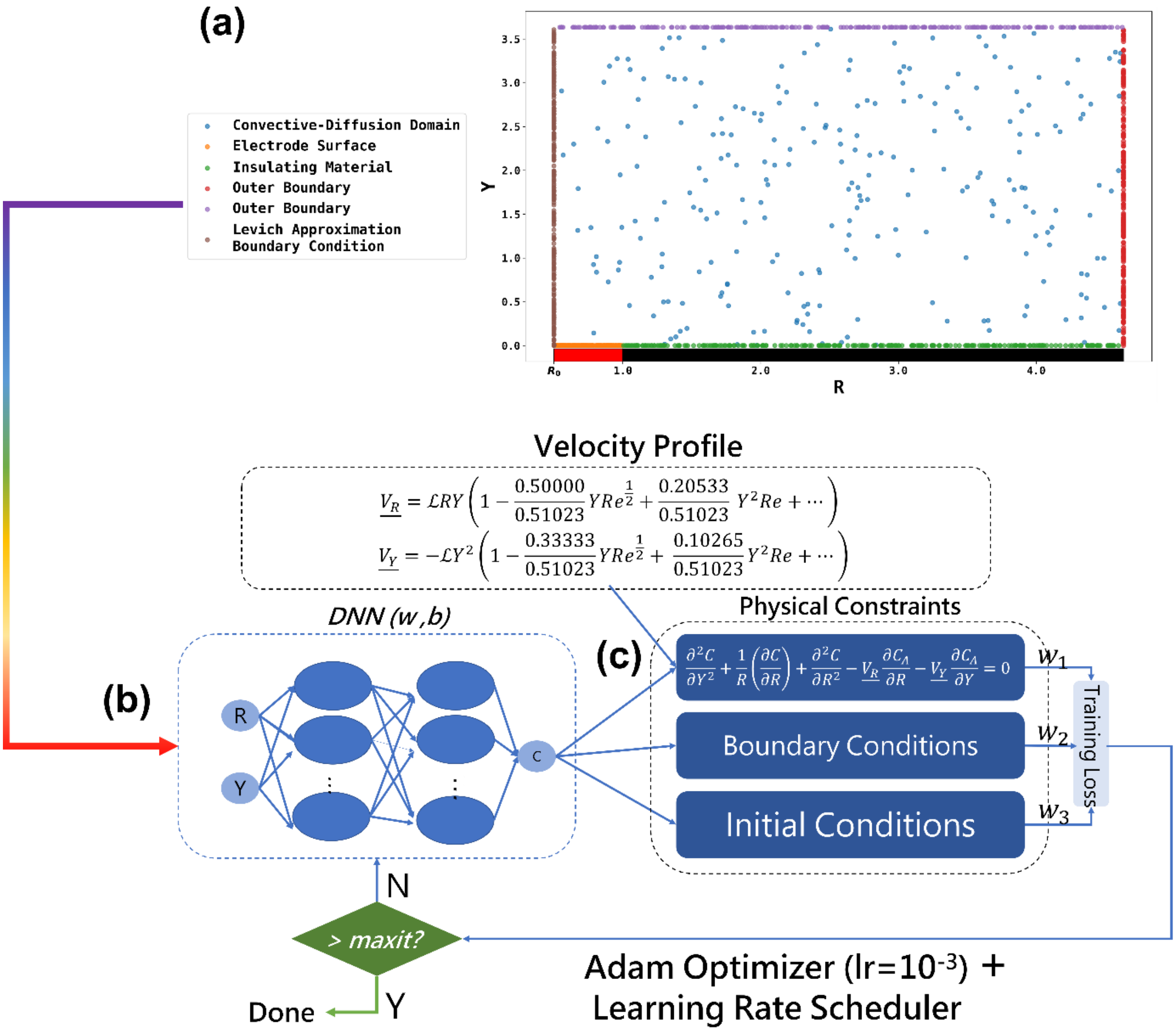

Figure S 2. The complete architecture of PINN simulation of 2-D steady-state simulation at a rotating disk electrode. (a) Collocation points are generated in the spatial domain corresponding to the convective-diffusion mass transport, initial and boundary conditions, then fed (b) into the neural network to predict the corresponding concentration while (c) satisfying the physical constraints.

6 Hyperparameter sensitivity

In this section, the sensitivity of two hyperparameters was tested. W_{sim}, in the context of 1-D simulation of cyclic voltammetry at a rotating disc electrode at a slow scan rate of 0.1V/s. As $W_{sim} = \Lambda W_{\delta}$, where W_{δ} is the dimensionless diffusion layer thickness and Λ is a multiple varying from 1 to 6 in this test. In Figure S 3, the voltammograms at different Λ are compared with FD simulation ground truth, suggesting that as long as $\Lambda > 2$, W_{sim} will approximate the semi-infinite boundary condition to offer a stable and robust simulation. In this paper, Λ was 6 for 1-D simulations, and $\Lambda = 4$ for 2-D simulations.

The second hyperparameter is R_0 in 2D simulation. R_0 determines the left boundary of simulation where Levich approximation as boundary condition is applied. R_0 is tested at 0.01,0.02, 0.05 and 0.1 to evaluate steady state flux as a function of dimensionless rotational frequency and shown in Figure S 4. The overlapping predictions at different R_0 suggest that the PINN simulation results are robust to the R_0 hyperparameter.

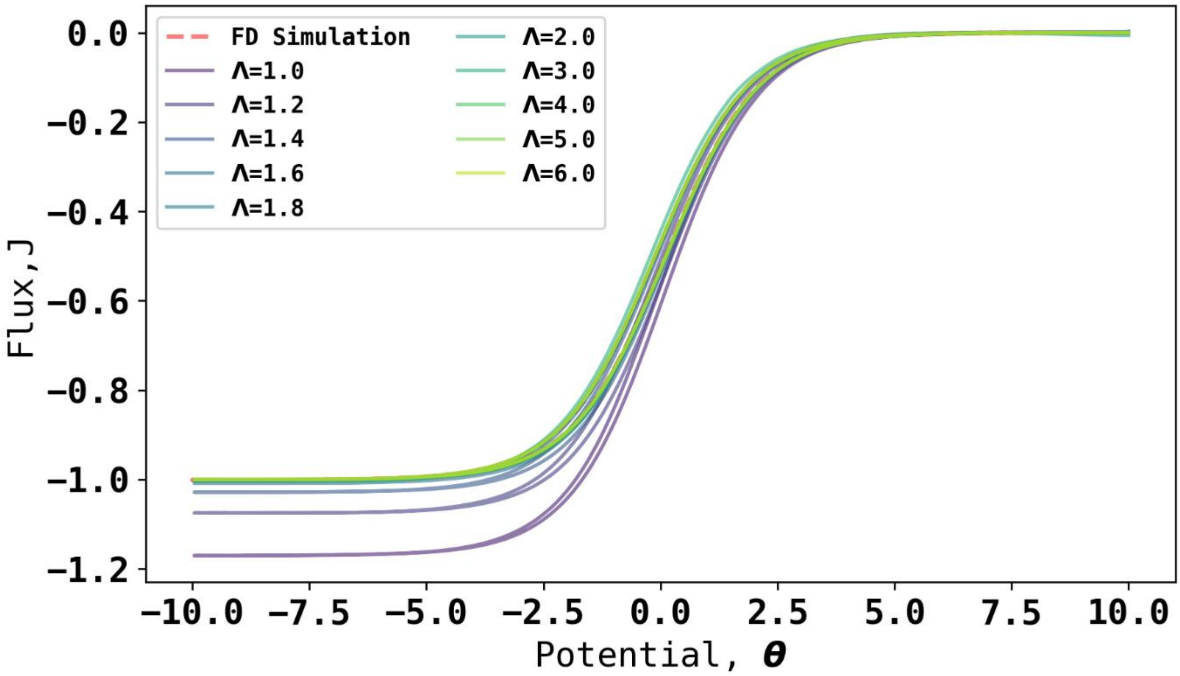

Figure S 3. PINN simulation of cyclic voltammetry at RDE as a function of Λ and compared with FD simulation ground truth (red dashed line). Λ the outer boundary of simulation relative to diffusion layer thickness.

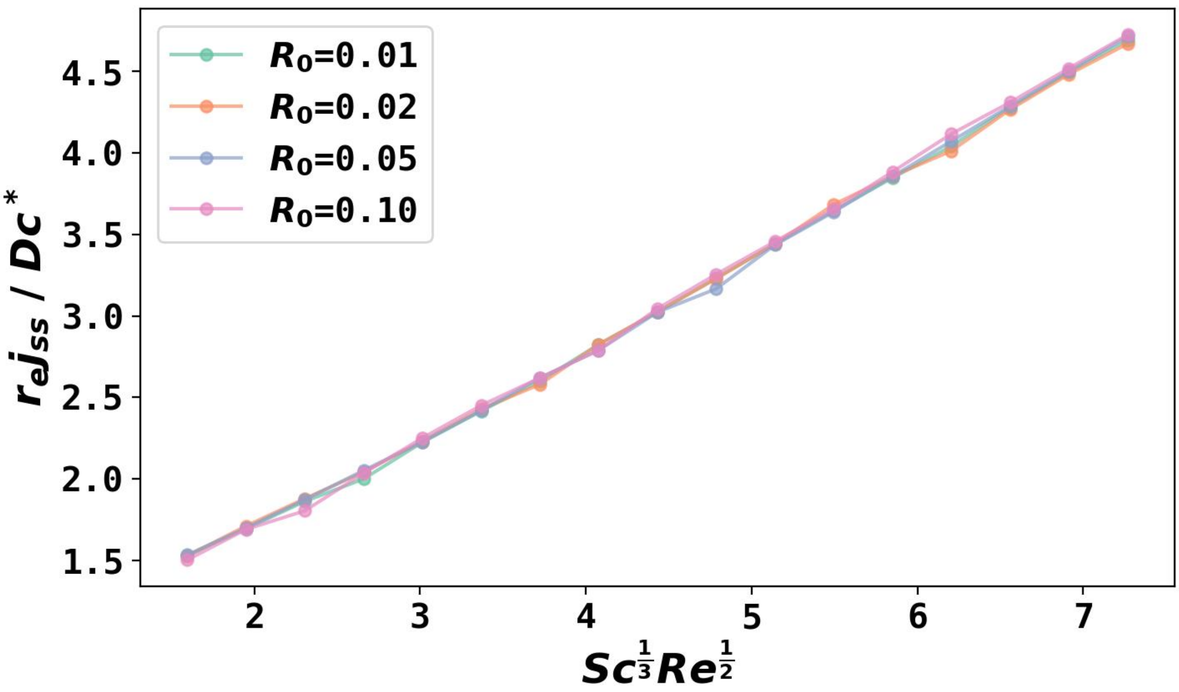

Figure S 4. 2-D simulation of steady state flux at RDE as a function of dimensionless rotational speed $Sc^{\frac{1}{3}}Re^{\frac{1}{2}}$ *at different* R_0.

7 Significance of the Edge Effect

To understand the significance of edge effect on a rotating disk electrode, edge effects are evaluated as a function of rotational frequency at different electrode radii ($100\ \mu m$ and 1 mm). The edge effect is determined significant if deviation from Levich equation is larger than 1%, where a critical rotational frequency is provided. Edge effects are more prominent below the critical frequency. A typical diffusion coefficient of $10^{-9}\ m^2 s^{-1}$ and kinematic viscosity of $10^{-6}\ m^2 s^{-1}$ are assumed in the conversion. Table S 2 illustrates the critical frequency at different electrode radius, suggesting that edge effect is significant experimentally if electrode size is at micrometre scale. The corresponding plots are shown Figure S 5 and Figure S 6.

Table S 2. The edge effect critical frequency as a function of electrode radius, below which the deviation from Levich will be greater than 1%.

Electrode Radius	Edge Effect Critical Frequency
$100\ \mu m$	$15.4\ Hz$
$1\ mm$	$0.15\ Hz$

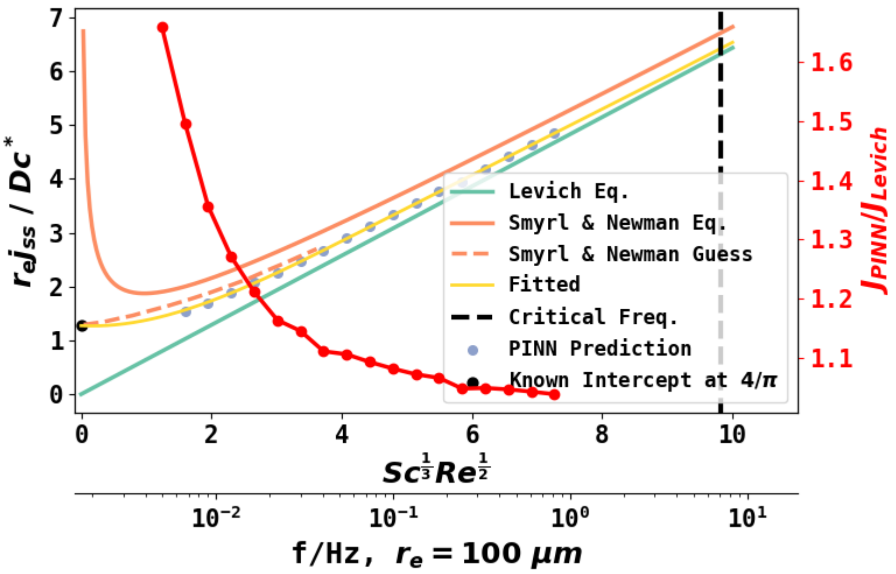

Figure S 5. Comparing the PINN prediction, Smyrl and Newman's work and the Levich equation prediction of steady state flux at different dimensionless rotation rates, $Sc^{\frac{1}{3}}Re^{\frac{1}{2}}$. PINN prediction was fitted along with known intercept at $\frac{\pi}{4}$ to give a general expression of peak current as $y = \frac{1}{1.544}x + 2.01\,exp(-0.35x) - 0.74\,exp(-0.07x^2)$. The right-hand y-axis and red line with markers present the ratio of PINN predicted current (with edge effect) relative Levich prediction. The lower x-axis provides guidance the edge effect as a function of rotational frequency when electrode radius is $100\ \mu m$. The black dashed line indicates the critical frequency below which the PINN predicted flux deviates more than 1% from Levich equation.

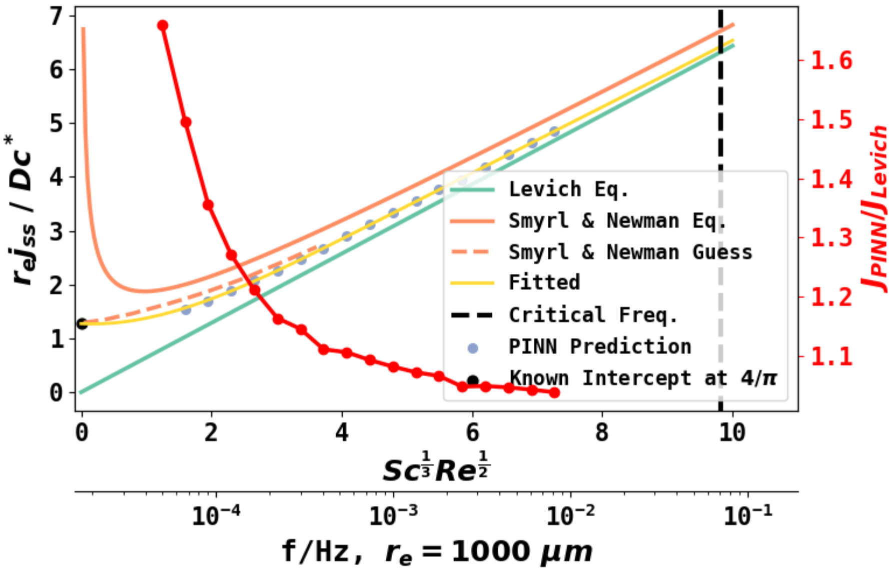

Figure S 6. Comparing the PINN prediction, Smyrl and Newman's work and the Levich equation prediction of steady state flux at different dimensionless rotation rates, $Sc^{\frac{1}{3}}Re^{\frac{1}{2}}$. PINN prediction was fitted along with known intercept at $\frac{\pi}{4}$ to give a general expression of peak current as $y = \frac{1}{1.544}x + 2.01\,exp(-0.35x) - 0.74\,exp(-0.07x^2)$. The right-hand y-axis and red line with markers present the ratio of PINN predicted current (with edge effect) relative Levich prediction. The lower x-axis provides guidance the edge effect as a function of rotational frequency when electrode radius is $1\ mm$. The black dashed line indicates the critical frequency below which the PINN predicted flux deviates more than 1% from Levich equation.

1. Britz, D.; Strutwolf, J., *Digital simulation in electrochemistry*. Springer: 2005; Vol. 666.
2. Gavaghan, D. J., How accurate is your two-dimensional numerical simulation? Part 1. An introduction. *J. Electroanal. Chem.* **1997,** *420* (1), 147-158.
3. Press, W. H.; Teukolsky, S. A.; Vetterling, W. T.; Flannery, B. P., *Numerical recipes 3rd edition: The art of scientific computing*. Cambridge university press: 2007.
4. Compton, R. G.; Laborda, E.; Kaetelhoen, E.; Ward, K. R., *Understanding voltammetry: simulation of electrode processes*. 2nd ed.; World Scientific London, 2020.
